# Case report: Partial regression of metastatic squamous cell carcinoma with altered azathioprine dosage after long-term use in renal transplant patient

**DOI:** 10.3389/fimmu.2024.1474663

**Published:** 2024-10-31

**Authors:** Tegan Ormston, Jessica Da Gama Duarte, Luke T. Quigley, Louise Jackett, John Whitlam, Andreas Behren, David E. Gyorki

**Affiliations:** ^1^ Department of Surgery, Austin Health, Heidelberg, VIC, Australia; ^2^ Olivia Newton-John Cancer Research Institute, School of Cancer Medicine, La Trobe University, Heidelberg, VIC, Australia; ^3^ Department of Pathology, Peter MacCallum Cancer Centre, Melbourne, VIC, Australia; ^4^ Kidney Transplant Service, Department of Nephrology, Austin Health, Heidelberg, VIC, Australia; ^5^ Department of Surgery, University of Melbourne, Parkville, VIC, Australia; ^6^ Victorian Clinical Genetics Services, Murdoch Children’s Research Institute, Royal Children’s Hospital, Parkville, VIC, Australia; ^7^ Department of Medicine, University of Melbourne, Parkville, VIC, Australia; ^8^Division of Cancer Surgery, Peter MacCallum Cancer Centre, Melbourne, VIC, Australia; ^9^Sir Peter MacCallum Department of Oncology, University of Melbourne, Melbourne, VIC, Australia

**Keywords:** case report, kidney transplant, immunosuppression, cutaneous squamous cell carcinoma, metastatic squamous cell carcinoma

## Abstract

**Introduction:**

We report the partial regression of metastatic squamous cell carcinoma (SCC) after reduction of long-term azathioprine therapy while awaiting surgery. The patient was a 69-year-old man with a history of kidney transplantation. Moderately differentiated SCC arising in the anterior neck was initially diagnosed, followed later by poorly differentiated SCC metastases to cervical lymph nodes. Lymph node clearance was performed 28 days after a reduction in azathioprine dosage. The palpable lymph node lesion had noticeably decreased in size at the time of surgery, and subsequent histology only detected 7mm and 0.2mm deposits of poorly differentiated SCC in 2 of 5 level I nodes, and a further 10 reactive nodes from levels II and III. One positive level I and another benign level II/III node, demonstrated necrosis, histiocytic infiltration and fibrosis, interpreted as features of regression. Hence, we investigated the role of immune cells in the partial regression of metastatic SCC after reduction of long-term azathioprine therapy while awaiting surgery.

**Methods:**

Multispectral immunohistochemistry using custom markers was performed on regions of interest of excised cervical lymph nodes, encompassing the entire SCC deposit and the surrounding adjacent stroma to quantify to number and types of immune cells present.

**Results:**

Multispectral immunohistochemistry revealed the heavy infiltration of activated T cells in the tumour, as well as PD-L1+ antigen-presenting cells in the surrounding adjacent stroma, suggesting an immunologically mediated partial regression.

**Discussion:**

We hypothesize that this reaction was triggered by azathioprine dose reduction. Dose modification of long-term immunosuppressive medications in patients with a transplantation history who later develop SCCs warrants further investigation.

## Introduction

An increased risk of malignancy is a known complication of organ transplantation and requisite immunosuppression. Overall, the incidence of cancers among transplant recipients is more than double that of the general population (1). Cancer is a major cause of death in transplant recipients (2). Cancers particularly associated with organ transplantation include non-melanomatous skin cancers (NMSC), immune deficiency and dysregulation-associated lymphoproliferative disorders including Hodgkin’s and non-Hodgkin’s lymphoma, anal cancer, multiple myeloma and Kaposi sarcoma (1). NMSCs, in particular cutaneous squamous cell carcinomas (cSCC), are among the most common cancers in these patients. Of note, treatment with azathioprine has been associated with higher rates of cSCC (3–5). This immunosuppressive agent increases the risk of cSCC via photosensitization of the skin to ultraviolet radiation, and the accumulation of 6-thioguanine within DNA, resulting in increased reactive oxygen species with UV exposure (3, 4). The outcomes for cSCC of the head and neck in transplant recipients are worse compared to immunocompetent patients, with higher rates of local or regional recurrence, and a lower disease-free survival, disease-specific survival, and overall survival (6). Spontaneous regression of metastatic cutaneous malignancy, particularly melanoma, is a well-documented phenomenon in immunocompetent patients (7). Similarly, a recent case report showed spontaneous regression of cSCC and in-transit metastases following cessation of ruxolitinib, a Janus kinase (JAK) inhibitor, which was used to treat postpolycythemia vera myelofibrosis (8). However, to our knowledge, this has not been described in an ongoing immunosuppressed patient.

## Case description

**Figure 1:**
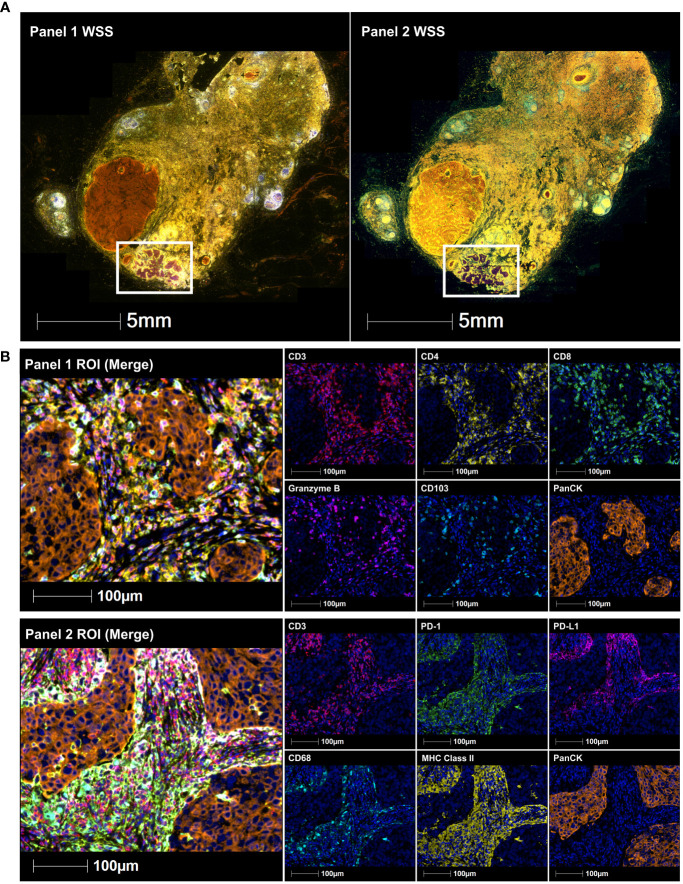
Immune-fluorescence images of a submandibular lymph node containing a 7mm deposit of poorly differentiated SCC. This includes **(A)** whole slide scans (WSS) and **(B)** Representative regions of interest (ROI) showing merge and single staining for panel 1: CD3 (red), CD4 (yellow), CD8 (green), granzyme B (magenta), CD103 (cyan), and panCK (orange) antibodies with DAPI (blue) counterstain (upper immune-fluorescence panel); and panel 2: CD3 (red), PD-1 (green), PD-L1 (magenta), CD68 (cyan), MHC class II (yellow) and panCK (orange) antibodies with DAPI (blue) counterstain (lower immune-fluorescence panel). All images were taken at 20x magnification, and scale bars indicate 100μm.

A 69-year-old man presented with self-detected cervical lymphadenopathy following a prior cSCC excision from the anterior neck. He had a history of kidney transplantation in 1980 from a blood group compatible brain-dead donor, tissue typing was not available for this patient. He was on long-term immunosuppression comprising azathioprine 75mg daily and prednisolone 5mg daily. Histology of the anterior neck skin tumour was a superficially invasive, moderately differentiated cSCC arising in a background of Bowen’s disease (SCC *in situ*). Both the *in situ* and invasive components had been completely excised with minimum 3 mm margin clearance. Seventeen months later the patient noticed a non-tender neck lump. Imaging workup with ultrasound and FDG-PET scan showed multiple abnormal left cervical lymph nodes in levels I (submental and submandibular group), II (upper jugular group) and III (middle jugular group), the largest of which was 26x17x21mm (**Figure 1A**). A core biopsy demonstrated poorly differentiated metastatic SCC, which was confirmed by immunohistochemistry (positive for AE1/AE3, CK5/6 and p63, and negative for S100). Haematoxylin and Eosin staining of this core biopsy demonstrated nests of viable SCC, some with associated brisk tumour infiltrating lymphocytes and some without (**Supplementary Figure**). During pre-operative work up for lymph node clearance, and one month following core biopsy, the patient’s azathioprine dose was reduced to 50mg alternating with 75mg daily. Prednisolone was continued at 5mg daily. He presented for lymph node clearance 28 days following the reduction of his azathioprine dose. At the time of surgery, clinical regression in the size of the palpable lymph node was noted.

**Figure 2:**
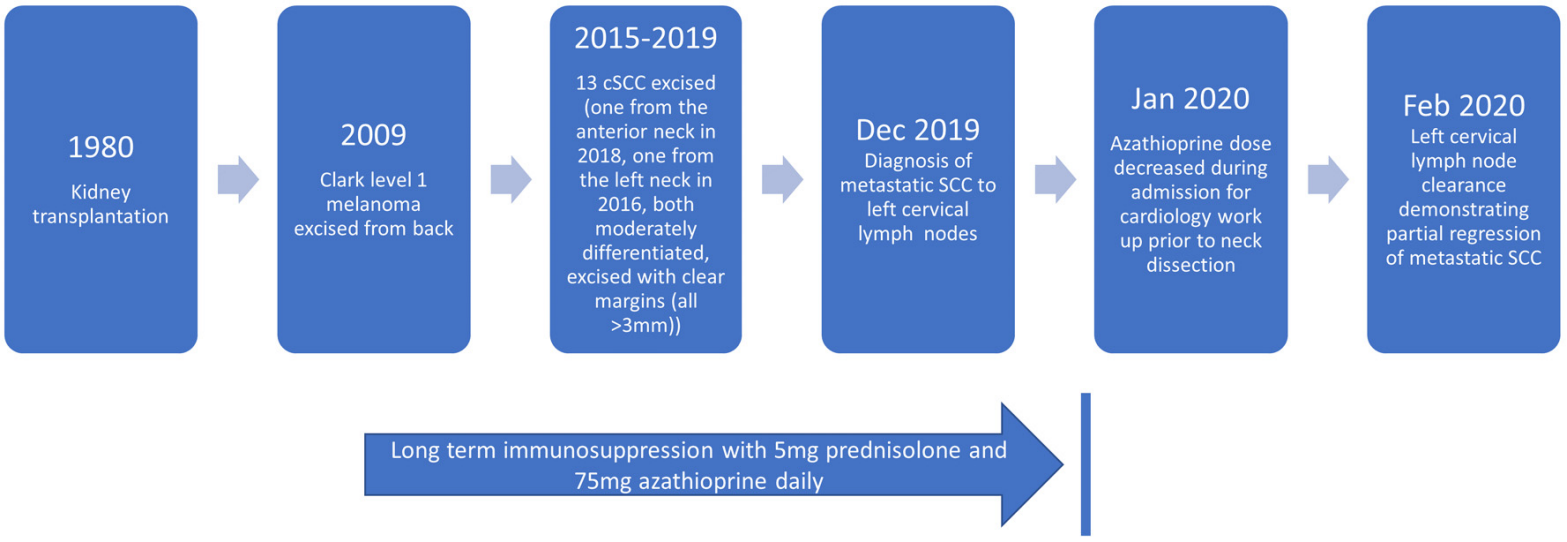
Clinical timeline detailing full patient history, and immunosuppressive medication.

Left sided lymph nodes (levels I-III) from the submandibular gland and parotid tail were submitted for formal histopathological examination. At level I, 2 of 5 lymph nodes were shown to be positive for metastatic SCC, both without extranodal extension. The largest deposit of viable tumour measured 7 mm, and this was located in a 22 mm diameter node that otherwise showed a background of extensive necrosis, fibrosis and histiocytic reaction. The second positive lymph node at level I (2 mm diameter) contained a 0.2 mm deposit of squamous cell carcinoma, without significant surrounding reaction. All nodes at levels II and III were negative for malignancy (total node count 0/10), however one of these nodes (12 mm diameter) demonstrated extensive fibrosis, neovascularization, and histiocytic infiltrate. The histological features of necrosis, inflammation and fibrosis in the above-described lymph nodes were interpreted as evidence of partial tumour regression. The patient currently remains in complete SCC remission, on 5mg prednisolone and 25mg azathioprine daily (please refer to **Figure 2** for clinical timeline). Azathioprine metabolite testing following azathioprine dose reduction found 6-methyl mercaptopurine (6-MMP) significantly elevated (1169 pmol per red blood cell, RBC) above the 6-thioguanine nucleotides (6-TGn) levels (below quantifiable limit), suggesting “shunting” variation in the activity of thiopurine methyl transferase (TPMT). Unfortunately, there is a paucity of literature looking at the impact of TPMT activity on the carcinogenic effects of azathioprine, with most literature focusing on the efficacy of azathioprine treatment and its toxicity (9).

Further analysis was undertaken on the resected level I lymph node that contained the 7mm deposit of poorly differentiated SCC (**Figure 1A**). Archival formalin-fixed paraffin-embedded (FFPE) tumour tissue of this node was interrogated for CD3, CD4, CD8, CD103, granzyme B, CD68, PD-1, PD-L1, MHC class II and pan-cytokeratin (panCK) expression using multispectral immunohistochemistry, as previously described (10). Primary antibody specifications included CD3 (SP7, ab16669, Abcam), CD4 (EPR6855, ab133616, Abcam), CD8 (C8/144B, MA5-13473, Invitrogen), CD103 (EPR4166 (2), ab129202, Abcam), granzyme B (D6E9W, 46890, Cell signaling), CD68 (PGM1, MA5-12407, Invitrogen), PD-1 (NAT105, ab52587, Abcam), PDL1 (E1L3N, 13684, Cell signaling), MHC class II (HLA-DR/DP/DQ/DX) (CR3/43, sc-53302, Santa Cruz) and panCK (AE1/AE3, Akoya Biosciences^®^). Slides were baked at 65°C for 2 hours, dewaxed in xylene three times for 10 min, rehydrated in ethanol twice for 10 min, and stained manually. The staining involved prior blocking of endogenous peroxidases using 3% hydrogen peroxide for 30 min, followed by sequential 15 min rounds of heat-induced epitope retrieval (microwave at 20% power), 10 min blocking of non-specific binding sites, 30 min primary antibody (CD3, CD4, CD8, CD103, granzyme B, CD68, Pd-1, PD-L1, MHC class II and panCK), 10 min secondary antibody (anti-mouse & anti-rabbit horseradish peroxidase) incubation, and 10 min fluorophore-tyramide signal amplification using Opal™ 520, 540, 570, 620, 650 and 690 fluorophores (Akoya Biosciences^®^) to detect all target proteins respectively. Slides were counterstained with spectral DAPI and scanned using the Vectra 3 Automated Quantitative Pathology Imaging System (Akoya Biosciences^®^). Images were spectrally unmixed using the inForm^®^ Cell Analysis software (Akoya Biosciences^®^) and analyzed using the HALO^®^ Image Analysis Platform (Indica Labs).

Tissue segmentation and cell phenotyping based on total DAPI^+^ cells was performed on regions of interest encompassing the entire SCC deposit and the surrounding adjacent stroma to quantify the number of total CD3^+^ T cells, CD4^+^ helper T cells, CD8^+^ killer T cells, CD3^+^PD-1^+^ T cells, CD3^+^CD8^+^granzyme B^+^ activated killer T cells, CD68^+^MHC class II^+^ PD-L1^+/-^ antigen-presenting cells, CD3^+^CD8^+^CD103^+^ tissue-resident memory T cells, CD3^+^CD8^+^CD103^+^granzyme B^+^ activated tissue-resident memory T cells and panCK^+^PD-L1^+/-^ tumour cells. The tumour microenvironment of the poorly differentiated SCC deposit was heavily infiltrated by T cells (16.5%), which included activated killer T cells (8.9%) and tissue-resident memory T cell (9.0%) subsets (**Figure 1B**, upper immune-fluorescence panel and cell counts). This was accompanied by the presence of PD-L1^+^ antigen-presenting cells, mainly in the surrounding adjacent stroma (4.8%) (**Figure 1B**, lower immune-fluorescence panel and cell counts), suggesting a highly immunoreactive microenvironment. Due to the abovementioned atypical changes seen in the remaining areas of this specimen, these findings could not be compared to adjacent normal lymph node regions.

## Discussion

Factors associated with increased risk of lymph node metastasis in cSCC include immunosuppression, clinical diameter of the primary lesion >2cm, increased thickness and poor differentiation of the primary lesion, and location of the primary lesion on the lip, ear, or posterior auricular region (11). In this case report, the patient’s only risk factor was immunosuppression. Despite the known poor outcomes in the setting of metastatic cSCC in solid organ transplant patients (11), our case demonstrates unexpected tumour regression, and a complete SCC remission.

Analysis of the tumour deposit and surrounding adjacent stroma of the resected level I lymph node suggests increased recruitment and abundance of tumour infiltrating T cells, killer T cells and activated killer T cells compared to the core biopsy specimen, in keeping with an active anti-tumour immune response and improved patient outcome (12). The T cell infiltrate also included a small proportion of mostly activated tissue-resident memory T (T_RM_) cells, the role of which is being increasingly investigated in tumour immunity (13–15). T_RM_ cells have recently been associated with poor clinical outcomes in primary cSCC lesions (16), and favorable outcomes in primary head and neck SCCs (13). However, their role in metastatic tissues is unclear. Notably, T_RM_ cells may either suppress or facilitate the spread of metastatic cells, based on cell heterogeneity and differing functions across tissues and diseases (17). In metastatic melanoma, T_RM_ cells have been found to mediate immunity in regional lymph nodes (15). However, identifying T_RM_ cells within lymph nodes can be complex (14), and their overall impact on metastatic cSCC patient outcomes warrants further investigation. Moreover, PD-L1^+^ antigen-presenting cells were seen in the adjacent stroma mainly surrounding the tumour, which may indicate the induction of previously reported antigen-specific immune tolerance or immune suppressive mechanisms (18). Azathioprine inhibits the replication of T and B cells by inhibiting purine synthesis, thus impacting RNA and DNA synthesis, while glucocorticoids exert their immunosuppressive function by downregulating antibody and complement binding, along with IL-2, IL-6, and IFN-γ production by T cells (19). We postulate that the decrease in azathioprine dose, however marginal, around the same time as the tumour disruption caused by the core needle biopsy allowed for the increase in tumour-reactive T cell populations.

Our observations suggest that the partial regression observed in our case was at least in part immunologically mediated, despite the presence of concurrent mechanisms of immune regulation or evasion. The effects of dose modification of long-term immunosuppressive medications in patients with transplantation history who later develop SCCs warrants further investigation.

## Data Availability

The original contributions presented in the study are included in the article/**Supplementary Material.** Further inquiries can be directed to the corresponding author.

## Supplementary material

Supplementary Figure 1 Haematoxylin and Eosin stain of core biopsy prior to the reduction in azathioprine dosage. Most (>95%) of the core biopsy is fibrosis **(B)**, with sparse nests of viable SCC **(A, C)** accounting for less than 5%. A couple of SCC nests are associated with brisk tumour infiltrating lymphocytes (TILs) **(A)**, while others are without TILs **(C)**. * denotes SCC nests.
