# Supplementary figures and images for: Case report: Partial regression of metastatic squamous cell carcinoma with altered azathioprine dosage after long-term use in renal transplant patient
Source: Front Immunol. 2024 Oct 31;15:1474663. doi: 10.3389/fimmu.2024.1474663 (PMC11561406; doi:10.3389/fimmu.2024.1474663)

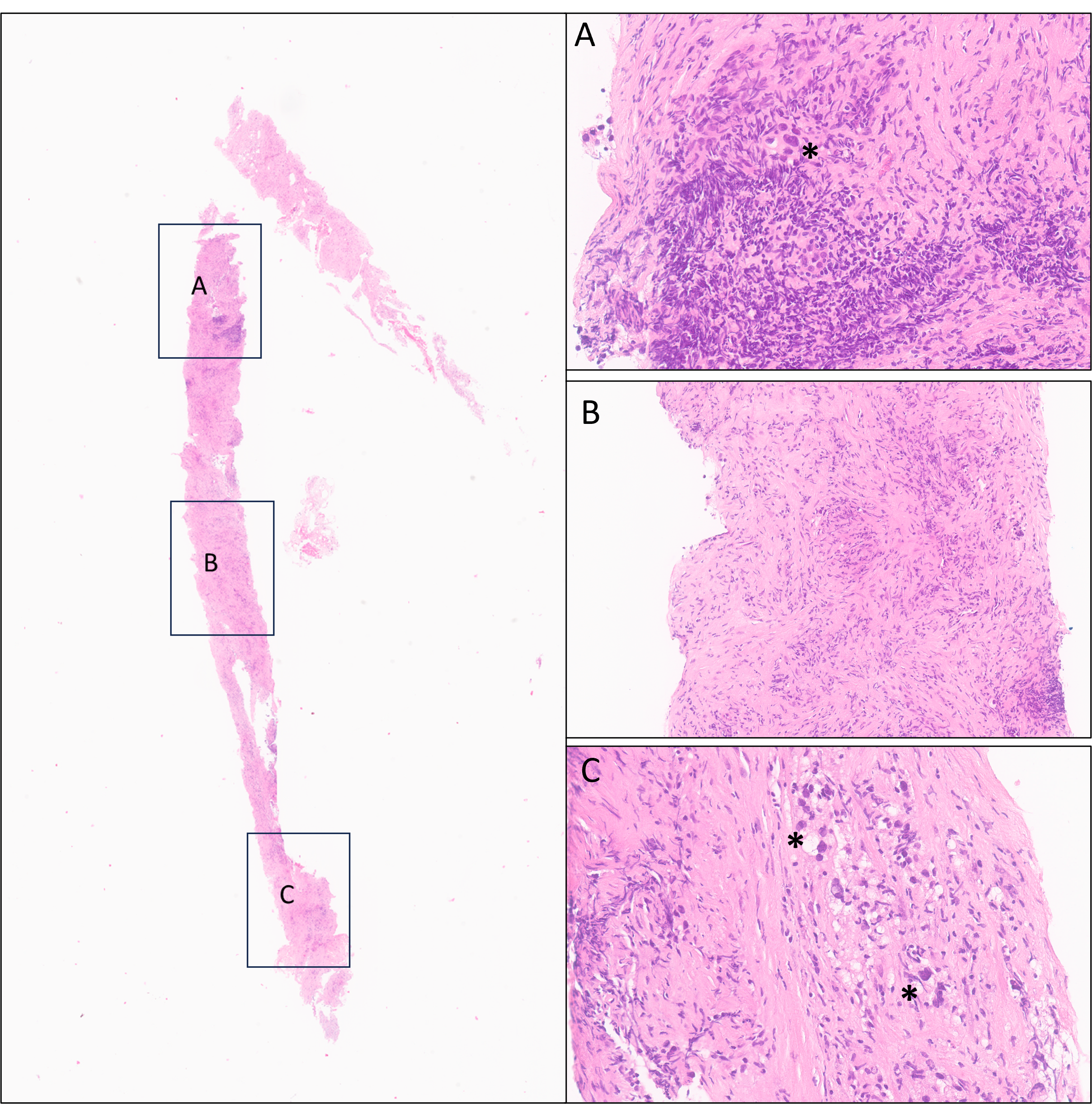

Supplement: Supplementary Figure 1 — Haematoxylin and Eosin stain of core biopsy prior to the reduction in azathioprine dosage. Most (>95%) of the core biopsy is fibrosis (B), with sparse nests of viable SCC (A, C) accounting for less than 5%. A couple of SCC nests are associated with brisk tumour infiltrating lymphocytes (TILs) (A), while others are without TILs (C). * denotes SCC nests. [file Image1.tiff]
